# TLTF in Cerebrospinal Fluid for Detection and Staging of *T. b. gambiense* Infection

**DOI:** 10.1371/journal.pone.0079281

**Published:** 2013-11-19

**Authors:** Maha-Hamadien Abdulla, Moiz Bakhiet, Veerle Lejon, Jan Andersson, James McKerrow, Omar Al-Obeed, Robert A. Harris

**Affiliations:** 1 Department of Surgery, Colorectal Research Center, King Khalid University Hospital, King Saud University, Riyadh, Kingdom of Saudi Arabia; 2 Department of Molecular Medicine, HH Princess Al-Jawhara Center for Genetics and Inherited Diseases – College of Medicine and Medical Sciences, Arabian Gulf University, Manama, Kingdom of Bahrain; 3 Department of Biomedical Sciences, Institute of Tropical Medicine, University of Antwerp, Antwerp, Belgium; 4 Departement of Infectious Medicine, Karolinska Institutet, Karolinska University Hospital Huddinge, Stockholm, Sweden; 5 Department of Pathology, Center for Discovery and Innovation in Parasitic Diseases, University of California San Francisco, San Francisco, California, United States of America; 6 Department of Clinical Neuroscience, Applied Immunology and Immunotherapy, Karolinska Institutet, Center for Molecular Medicine, Karolinska Hospital in Solna, Stockholm, Sweden; Charité, Campus Benjamin Franklin, Germany

## Abstract

**Background:**

Trypanosome-derived lymphocyte triggering factor (TLTF) is a molecule released by African trypanosomes that interacts with the host immune system, resulting in increased levels of IFN-γ production.

**Methodology/Principal findings:**

TLTF and anti-TLTF antibodies were assessed in sera and cerebrospinal fluid (CSF) from patients infected with *Trypanosoma brucei gambiense* (*T. b. gambiense*) in an attempt to identify alternative markers for diagnosis and stage determination of human African trypanosomiasis or sleeping sickness. Seventy-four serum and sixty-one CSF samples from patients with parasitologically confirmed infection and known disease stage along with 13 sera and CSF from uninfected controls were tested. In serum the levels of anti-TLTF antibodies were unrelated to the disease stage. In contrast, levels of anti-TLTF antibodies in CSF were higher in intermediate/late stages than in early stage disease patients. Specificity of the detected antibodies was assessed by inhibition of TLTF bioactivity as represented by its ability to induce IFN-γ production. Additionally, TLTF was detected in CSF from late stage patients by Western blotting with the anti-TLTF specific monoclonal antibody MO3.

**Conclusions/Significance:**

These findings suggest a new possibility for disease diagnosis with focus on involvement of the CNS through detection of TLTF and anti-TLTF antibodies in the CSF.

## Introduction

African trypanosomes are major pathogens of humans and livestock. The pathogen is transmitted by the bite of infected tsetse flies (*Glossina sp.*) and multiplies extracellularly in the blood and tissue fluids of the human host. Two subspecies of *Trypanosoma brucei* (*T. b. rhodesiense* and *T. b. gambiense*) cause human African trypanosomiasis (HAT, commonly called sleeping sickness). After replicating at the tsetse fly bite site trypanosomes enter the hemolymphatic system (early stage or stage 1) [1], [2]. Without treatment the parasites invade the central nervous system (CNS; late stage or stage 2), a process that takes months-to-years with *T. b. gambiense* (West and Central African HAT) or weeks-to-months with *T. b. rhodesiense* (East African HAT). The parasites cause a meningoencephalitis leading to progressive neurological involvement with concomitant psychiatric disorders, fragmentation of the circadian sleep-wake cycle and ultimately to death if untreated [1], [2], [3]. Currently over 60 million people living in 36 sub-Saharan countries are at risk of contracting the disease [4], [5], [6]. Due to reinforced surveillance the number of new cases reported in 2009 had fallen below 10,000 for the first time in 50 years. In 2010 the estimated number of new cases was thought to be approximately 7139. [7].

A key issue in the treatment of HAT is to distinguish stage 1 disease from stage 2 disease, as the drugs used for the treatment of stage 2 need to cross the blood-brain barrier [8], [9]. The most widely used drug is melarsoprol (developed in 1949), which is effective for *T. b. gambiense* and *T. b. rhodesiense* HAT, but unfortunately melarsoprol leads to severe and fatal encephalitis in about 5–10% of recipients despite treatment for this condition [10], [3], [1]. Where HAT is endemic accurate staging is therefore critical, because while failure to treat CNS involvement leads to death, inappropriate CNS treatment unnecessarily exposes an early-stage patient to highly toxic and life-threatening drugs. The diagnosis of HAT in the rural clinical setting, where most patients reside, still largely relies on the detection of parasitaemia by blood smear and/or CSF microscopy [11], [12].

Experimental studies have revealed that *T. b. brucei* releases trypanosome-derived lymphocyte triggering factor (TLTF), triggering CD8^+^ T cells to secrete IFN-γ in a non-antigen-specific manner [13], [14]. The action of TLTF is not host species restricted since both rat and human mononuclear cells can be activated to secrete IFN-γ. TLTF is conserved within the Trypanozoon subgenus, including the human infective *T. b. gambiense* and *T. b. rhodesiense* [15], and both TLTF and anti-TLTF antibodies can be detected in mice infected with *T. b. brucei* [16]. Considering TLTF as a trypanosome-specific molecule we investigated its potential for serodiagnostic purposes in HAT. We describe TLTF and anti-TLTF antibody detection in paired serum and CSF of patients with *T. b. gambiense* HAT.

## Materials and Methods

### Ethical statement

The study was approved by the National Ethical Committee of the Ministry of Health of the Democratic Republic of Congo (D.R.C.). HAT patients gave written informed consent before enrolment. Children (<18 years) or patients with altered mental status, a common condition in late stage HAT, were only included after written informed consent from a parent or a guardian. All patients had the option of withdrawing from the studies at any time.

### Patients

Seventy-four serum and sixty-one CSF samples were collected from patients in the Democratic Republic of Congo. They were obtained for routine diagnostic purposes from parasitologically confirmed *T. b. gambiense* patients before treatment during sleeping sickness control activities. White blood cell (WBC) counts and presence of trypanosomes in CSF were assessed in the hospital of Bwamanda for stage determination. Storage was at −20°C in the D.R.C. and at −70°C in Europe. Patients did not undergo systematic screening for co-infections.

Patients were classified according to WHO criteria. The upper limit for normal and cut-off values for the haemolymphatic stage has been set at 5 WBC/microliter [17]. Patients with values between 5–20 WBC/microliter are considered in the intermediate stage. A WBC count >20 WBC/microliter or the presence of trypanosomes in the cerebrospinal fluid (CSF) indicates the meningo-encephalitic stage. Twenty-five patients were in the Early (E) stage, 25 patients in Intermediate (I) and 24 patients in the Late (L) stage. Six control serum and 13 control CSF samples originated from Swedish Multiple Sclerosis patients attending the Neurology Clinic at Karolinska Hospital, Sweden.

### TLTF preparations

Recombinant TLTF (rTLTF) was prepared as described elsewhere [18]. Based on previous studies native TLTF (nTLTF) was prepared as follows. Monomorphic trypanosomes *T. b. brucei* AnTat 1.1 were harvested 6 days post-infection from rats by DEAE chromatography [19], [20]. A 10^6^/ml trypanosome suspension was incubated with 100 U/ml rat IFN-γ for 1 hour at 37°C. The supernatant was clarified by centrifugation at 12,000 g for 5 min before ultracentrifugation using JumboSep 100 kDa cut-off filtration devices (Pall Gelman). The concentrated supernatant was loaded onto a MONO Q ion exchange column (Pharmacia) in 50 mM Tris pH 7 and eluted with the same buffer containing 1M NaCl using an FPLC system (Pharmacia). Eluted peaks were collected separately, run in 10% SDS PAGE gels and silver stained. TLTF was stored at 4°C.

### Anti-TLTF ELISA

An ELISA was used to detect antibodies against TLTF in patient sera and CSF, respectively. Flat-bottom 96-well polystyrene plates (polysorp F96, Nunc, Glostrup, Denmark) were coated with 100 µl 10 ng/ml rTLTF in bicarbonate buffer pH 9.6 overnight at room temperature (RT). Control wells were coated with 100 µl of 10 ng/ml ovalbumin in bicarbonate buffer pH 9.6. Wells were washed five times with 200 µl phosphate-buffered saline (PBS) containing 0.05% Tween-20 (PBS-T) and saturated with 100 µl 1% bovine serum albumin (BSA) in PBS for 1 h at RT. After washing the plates with 200 µl PBS-T, specimens were dispensed into TLTF and ovalbumin coated wells in 100 µl amounts at a 1:100 dilution in PBS for CSF and 1:1000 dilution for serum. These dilutions were selected after testing several other dilutions. After 1 h incubation at 37°C and five washes with 200 µl PBS-T, 100 µl of biotinylated goat anti-human IgG (Sigma, St Louis, MO, USA) diluted 1:1,000 in PBS were added for 1 h at 37°C. One hundred µl of avidin-biotin-alkaline phosphatase conjugate (ABC-AP; Vector Laboratory, Burlingame, USA) diluted 1:100 in PBS were added for 45 min. Five consecutive washings with PBS removed unbound ABC-AP, and 100 µl of freshly prepared enzyme substrate solution was added per well. Absorbance was measured at 405 nm in a Multiscan photometer (mcc/340; Lab system, Helsinki, Finland). For each specimen, a corrected optical density (O.D.) was calculated.

### Western blot for anti-TLTF detection

To confirm the specificity of anti-TLTF antibodies 8 µg/well nTLTF was run in non-denaturing, 10% SDS PAGE preparative gels, transferred to nitrocellulose membranes (Amersham) for 1 hour at 100 V and blocked with 5% low fat milk in PBS-T for 1 h at RT. The membranes were placed into a 20-channel Mini-PROTEAN II Multiscreen apparatus (Bio-Rad, Sollentuna, Sweden) and the channels were filled for 1 h at RT with 400 µl of a 1:100 dilution of CSF with 5% low fat milk in PBS-T as a reagent blank. After three washes with 500 µl PBS-T the membrane was removed and incubated with 5 ml mouse anti-human IgG horseradish peroxidase (HRP) conjugate (Amersham International, Uppsala, Sweden) diluted 1:1,000 in 5% low fat milk/PBS-T for 1 h at RT. After washing three times with 50 ml PBS-T, substrate and chromogen solution was added to visualize bound IgG (ECL system, Amersham). As positive control, similar blots were run with 400 µl of 5 µg/ml MO3/channel and developed with rabbit anti-mouse IgG-peroxidase conjugate. Negative controls consisted of the same blots without human specimen and without MO3.

### Inhibition of TLTF bioactivity by CSF in ELISPOT

The ELISPOT method described and adapted to mouse IFN-γ was used to detect inhibition of IFN-γ production by single secretory cells [21], [22]. Naive DBA/1 mice spleens were dissected, splenocytes purified and viable cells were diluted to 2×10^6^/ml in RPMI. The assay was performed in nitrocellulose bottomed 96-well microtiter plates (Multiscreen HA; Millipore, Bedford, UK) using 15 µg/ml IFN-γ specific mouse monoclonal antibody DB1 [23] and 200 µl/well (4×10^5^ cells) of the above prepared cell suspension. Twenty µl of native TLTF (nTLTF) were added to triplicate wells at a concentration of 8 µg/well, with none being added to unstimulated wells. Patient and control CSF samples were diluted 1:10 in PBS and pre-absorbed with of 8 µg/ml nTLTF for 30 min at RT. Twenty µl volumes of pre-adsorbed samples were added in triplicate to the wells. Con-A was used as positive control and similarly preabsorbed with patients CSF to examine the specificity of the anti-TLTF antibodies.

Cells were cultured for 24 h before sequential incubation with polyclonal rabbit anti-rat IFN-γ antibody, 1:1000 swine anti-rabbit biotinylated antibody and colour development with avidin-biotin-alkaline phosphatase conjugate (ABC-AP; Vector Laboratory, Burlingame, USA) and 3-amino-9-ethycarbazole and H_2_O_2_ [24]. The spots were counted in an automatic counter (TransTec) and the mean value of the triplicates calculated for each sample.

### Detection of TLTF in the CSF by Western blotting

CSF samples were diluted 1:100 5% low fat milk in PBS-T and 20 µl of the non- denatured samples was added to each lane and run in 10% SDS PAGE gels to test the presence of TLTF in the samples. CSF samples from two patients in each stage were run in parallel with the controls, MO3 was diluted at 5 µg/ml and analysis by Western blotting conducted as before.

### Statistical analysis

The non-parametric Mann-Whitney test was used to evaluate statistical differences.

## Results

### Anti-TLTF antibodies in sera and CSF determined by ELISA

**Figure 1:**
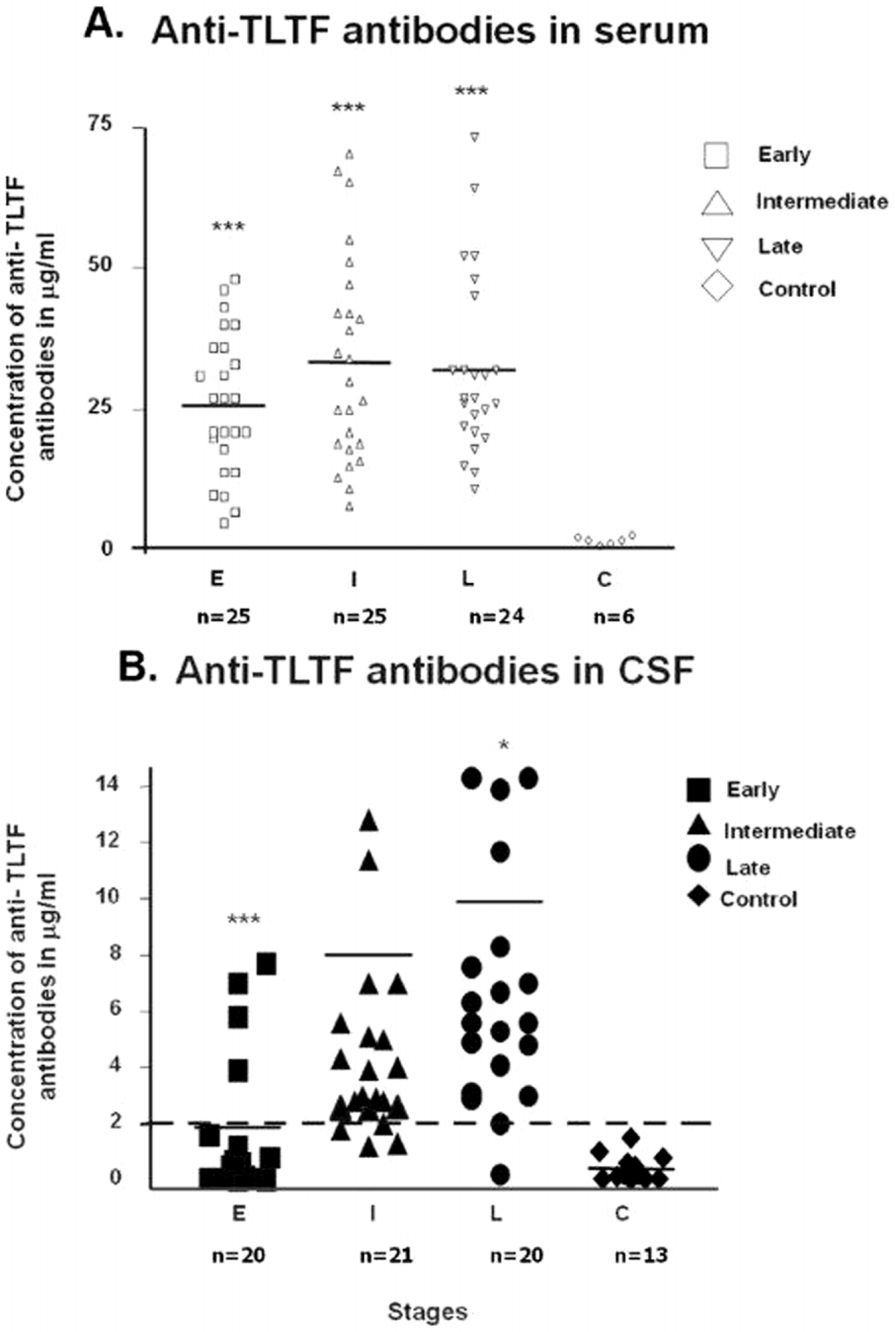
Anti-TLTF antibodies in sera and CSF determined by ELISA. (a). Concentrations obtained in anti-TLTF ELISA in serum samples of early stage (E, n = 25), intermediate stage (I, n = 25) and late stage (L, n = 24) patients. Differences between E, I and L are not significant from each other but are significant compared to the control (C) samples (p = 0.0002). (b). Concentrations of anti-TLTF in CSF samples of early stage (E, n = 20) patients, intermediate stage (I, n = 21) and late stage (L, n = 20) patients. Note the significant difference between intermediate and early stage (*P = 0.0005*) and between intermediate and late stage (*P<0.05*). The levels of TLTF in CSF are represented in (µg/ml). The horizontal hatched line marks the diagnostic cut-off value (2 µg/ml).

Concentrations of anti-TLTF antibodies were obtained in serum, CSF and control samples. The control sera gave low absorbance levels. Anti-TLTF antibodies were detected in the sera of all HAT patients. There was no significant difference between the mean concentrations of each HAT group but mean concentrations obtained for each of these groups differed significantly (*p = 0.0002*) from the mean concentration obtained with the control sera (Fig. 1 A).

In CSF, anti-TLTF antibodies were only detectable in the intermediate and the late stage groups with no significant difference (*p = 0.029*) of the mean concentrations between either of these groups. In the early stage group and the control sera the mean concentrations obtained were significantly lower (*p<0.001*) than in intermediate and late stage CSF and were thus considered negative for anti-TLTF antibodies (Fig. 1B).

### TLTF is detectable in CSF of late stage sleeping sickness patients

**Figure 2:**
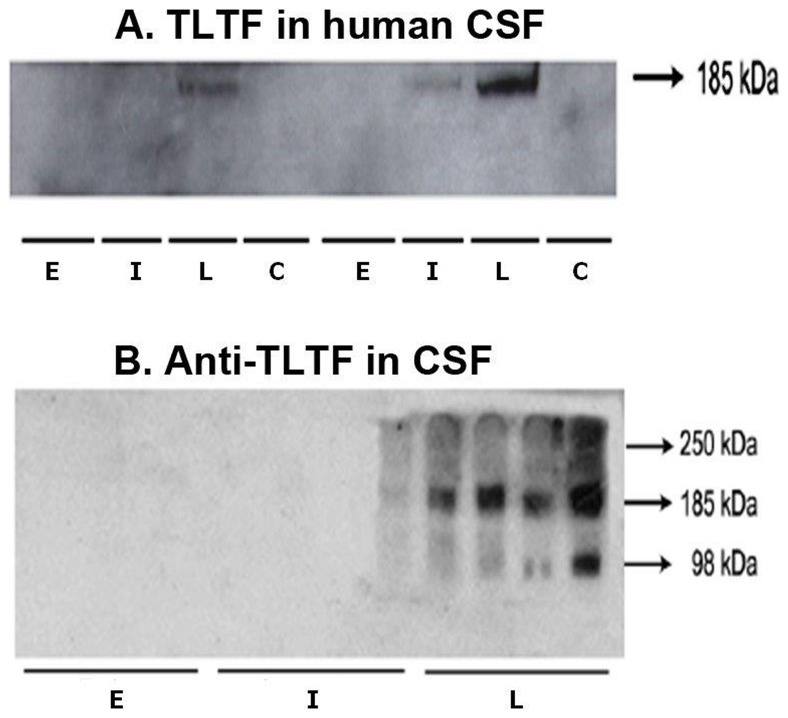
Specificity of CSF anti-TLTF antibodies for native TLTF in Western blot. (a). Immunoblots to detect the presence of TLTF in CSF from HAT patients and controls. The anti-TLTF monoclonal antibody MO3 was used as a detection antibody and the presence of TLTF in the CSF samples was revealed as a band with a MW of approximately 185 kDa. (b) Immunoblots using CSF samples from each stage of the disease to detect the occurrence of anti-TLTF antibodies in CSF. Native TLTF (nTLTF) was used as an antigen in these experiments and revealed as a band with a molecular weight of approximately 185 kDa.

After running CSF samples using Western blotting and an anti-TLTF monoclonal MO3 as primary antibody, a 185 kDa band indicative for TLTF was only detected in samples from late stage patient CSF (Figure 2 A).

### Specificity of CSF anti-TLTF antibodies for native TLTF in Western blots

To test the specificity of CSF anti-TLTF antibodies detected by ELISA non-denaturing Western blotting was performed with nTLTF and 4 CSF samples from each HAT group (Figure 2 B). Under these conditions TLTF appears as a single band at 185 kDa [3] and was apparent in all 4 samples in late stage patients and in one intermediate stage patient sample. MO3 used as a positive control detected the same 185 kDa molecular weight band (data not included). A smaller MW band may be a breakdown product of TLTF. No bands were revealed in samples from the other stages.

### Inhibition of nTLTF bioactivity by CSF of sleeping sickness patients

**Figure 3:**
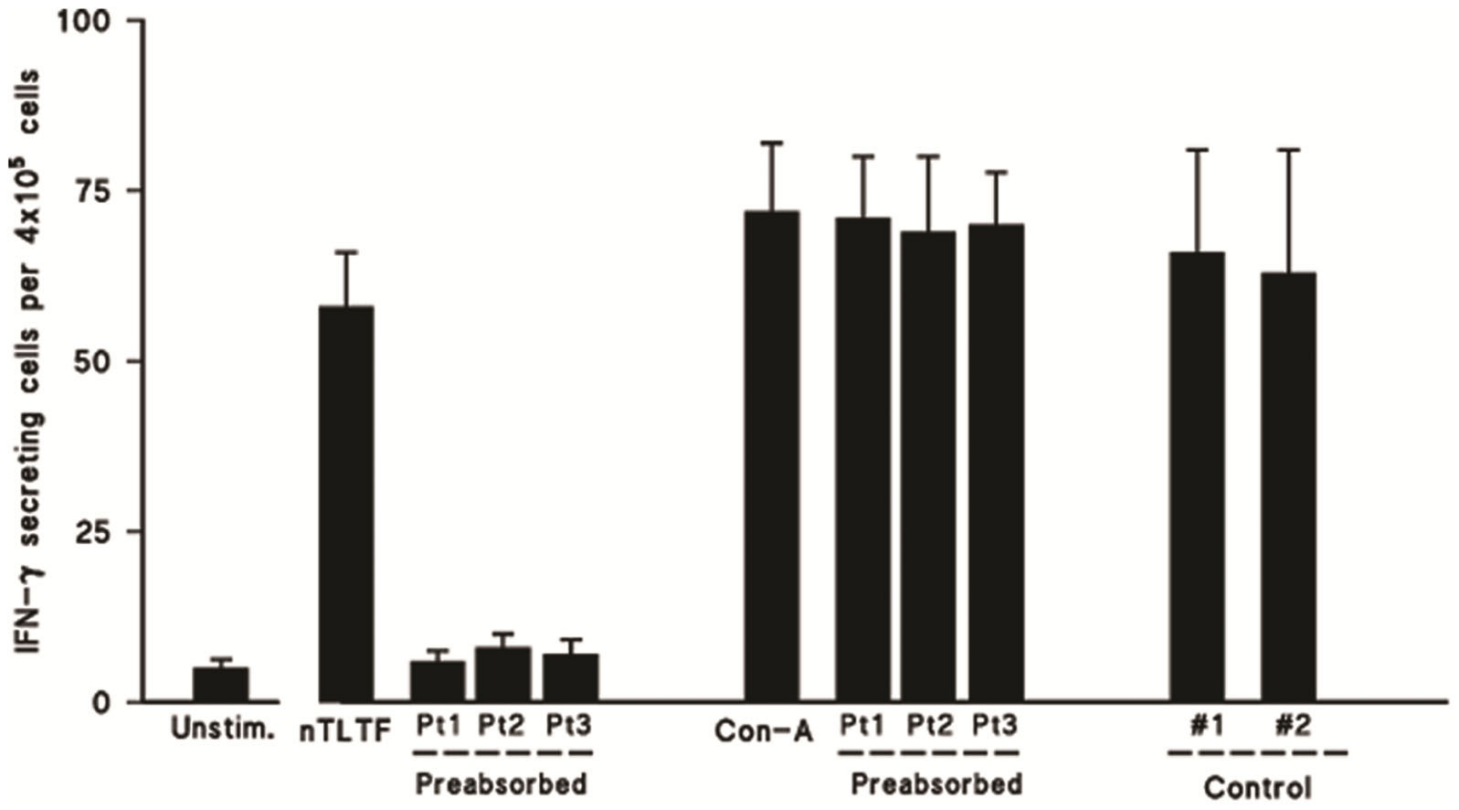
Inhibition of nTLTF bioactivity by CSF of sleeping sickness patients. Effect of preabsorption of 8 µg native TLTF (nTLTF) by anti-TLTF antibodies in CSF samples from 3 patients and controls. Inhibition of nTLTF activity is reflected by a decreased number of IFN-γ secreting cells compared to the effect of nTLTF alone (*P<0.05*). Note the absence of preabsorption effects by anti-TLTF antibodies in the CSF samples on Con-A stimulated IFN-γ production, suggesting the specificity of the anti-TLTF antibodies detected in the CSF from the 3 patients.

To further investigate the specificity of anti-TLTF antibodies detectable in CSF by ELISA and Western blotting, inhibition of TLTF bioactivity assay was assessed by ELISPOT (Figure 3). The number of IFN-γ secreting cells stimulated with TLTF was 56+/−18 (mean+/−SD) compared to those that did not receive TLTF (mean 5+/−1.2). The same stimulatory effect was observed when TLTF was mixed with control CSF samples before being added to the cells (mean 60+/−38). The numbers of IFN-γ secreting cells that received pre-adsorbed CSF samples of HAT patients were significantly lower than in the TLTF-stimulated control wells (p<*0.005*) and were comparable to unstimulated cells. Con-A was used as positive control and yielded significantly higher induction of IFN-γ compared to unstimulated cells (p<*0005*). This action of ConA was not inhibited by anti-TLTF antibodies in the CSF.

## Discussion

TLTF is a glycoprotein released by bloodstream form trypanosomes from the *Trypanozoon* subgenus, resulting in CD8^+^ T lymphocyte activation and production of IFN-γ. Experimental studies have demonstrated *in vivo* production of TLTF during infection and generation of anti-TLTF antibodies, correlation of parasite load with the ability of TLTF to stimulate CD8^+^ T cells, and active production of TLTF by living parasites in response to IFN-γ [16], [19]. A role for TLTF in HAT has not yet been demonstrated. In this report we assessed the usefulness of TLTF and anti-TLTF antibodies as a HAT biomarker, with focus on measurement in CSF.

Anti-TLTF antibodies were detected using ELISA in the serum of all HAT patients, the antibody levels being independent of clinical stage of the disease. This indicates that TLTF is released by *T. b. gambiense* bloodstream forms from early on in the infection in sufficient amounts to induce a humoral immune response. In the CSF samples antibodies were only detectable in HAT patients with evidence of trypanosome invasion of the central nervous system (I and L stages), suggesting TLTF release by intrathecally resident parasites and subsequent intrathecal antibody production.

The specificity of the anti-TLTF antibodies in CSF was confirmed through Western blotting using a selection of the samples. Purified native TLTF recognized by the specific monoclonal MO3 was revealed in the same way by the late stage CSF samples and some intermediate stage samples, but not by the early stage samples. Further specificity confirmation evident in the ELISPOT indicates that the production of IFN-γ by mouse splenocytes induced by nTLTF is inhibited when nTLTF is first incubated with CSF samples containing anti-TLTF antibodies, but this was not the case when Con-A was used as a positive control.

Earlier attempts to detect TLTF in serum of HAT patients using capture ELISA were unsuccessful. This can be explained by the characteristically low parasitemia of *T. b. gambiense* in HAT patients, presumably resulting in release of TLTF amounts that remain below the detection limit of the assay. Alternatively, TLTF might be present but in complex with antibodies or by its target receptor (CD8) and thus unavailable for the capturing monoclonal antibody in non-denaturing ELISA conditions.

While the detection of TLTF in CSF implicates a role for its action within the CNS, the presence and inhibitory character of anti-TLTF antibodies in serum and CSF indicates a humoral immune response that might interfere with the infection course. In this respect it would be interesting to study TLTF and anti-TLTF profiles in biological samples of HAT patients infected with *T. b. rhodesiense*. HAT caused by this parasite usually causes a much more fulminant disease with high parasitemia leading to death within a few months, in contrast with *T. b. gambiense* that causes a chronic disease with death occurring after several months or some years [25].

We cannot entirely exclude that other infections than trypanosomiasis might have had an impact on potential brain inflammation/complications that could subsequently affect clearance/penetration of trypanosome-derived macromolecules. Co-infections in the African continent may be a common event, and their immunological consequences have been reported in a number of settings [26]–[31]. Thus the production of an immunomodulatory substance by trypanosome parasites is not unique, as exemplified by eicanosoids during intracellular bacterial infections [32] and excretory-secretory filarial substances [33], [34]. This is thus an issue that would be worthy of further attention, as it is possible that the synergistic immunomodulatory effects of co-infections impacts on the type and severity of pathology of a given parasite infection.

In conclusion, this work demonstrates a possible role for TLTF in *T. b. gambiense* HAT pathogenesis. In addition, the profiles presented here of anti-TLTF antibodies in CSF of HAT patients opens perspectives for the development of alternative tests for both diagnosis and differentiation of early versus intermediate/late stage infections. Further work will be required to determine the biological meaning and other possible applications of anti-TLTF antibodies in CSF.
